# EPSILoN: A Prognostic Score for Immunotherapy in Advanced Non-Small-Cell Lung Cancer: A Validation Cohort

**DOI:** 10.3390/cancers11121954

**Published:** 2019-12-05

**Authors:** Arsela Prelaj, Roberto Ferrara, Sara Elena Rebuzzi, Claudia Proto, Diego Signorelli, Giulia Galli, Alessandro De Toma, Giovanni Randon, Filippo Pagani, Giuseppe Viscardi, Marta Brambilla, Benedetta Trevisan, Monica Ganzinelli, Antonia Martinetti, Rosaria Gallucci, Rosa Maria Di Mauro, Giuliano Molino, Nicoletta Zilembo, Valter Torri, Filippo Maria de Braud, Marina Chiara Garassino, Giuseppe Lo Russo

**Affiliations:** 1 Medical Oncology Department, Fondazione IRCCS Istituto Nazionale Tumori, 20133 Milan, Italy; roberto.ferrara@istitutotumori.mi.it (R.F.); claudia.proto@istitutotumori.mi.it (C.P.); diego.signorelli@istitutotumori.mi.it (D.S.); giulia.galli@istitutotumori.mi.it (G.G.); alessandro.detoma@istitutotumori.mi.it (A.D.T.); giovanni.randon@istitutotumori.mi.it (G.R.); filippo.pagani@istitutotumori.mi.it (F.P.); giuseppe.viscardi@istitutotumori.mi.it (G.V.); marta.brambilla2@istitutotumori.mi.it (M.B.); bene.trevisan@gmail.com (B.T.); monica.ganzinelli@istitutotumori.mi.it (M.G.); antonia.martinetti@istitutotumori.mi.it (A.M.); rosaria.gallucci@istitutotumori.mi.it (R.G.); rosa.dimauro@istitutotumori.mi.it (R.M.D.M.); giuliano.molino@istitutotumori.mi.it (G.M.); nicoletta.zilembo@istitutotumori.mi.it (N.Z.); Filippo.DeBraud@istitutotumori.mi.it (F.M.d.B.); marina.garassino@istitutotumori.mi.it (M.C.G.); Giuseppe.LoRusso@istitutotumori.mi.it (G.L.R.); 2 Medical Oncology Unit 1, IRCCS Ospedale Policlinico San Martino, Largo Rosanna Benzi 10, 16132 Genova, Italy; saraelena89@hotmail.it; 3 Pharmacological Research Institute IRCSS Mario Negri, Via La Masa 19, 20156 Milan, Italy; valter.torri@marionegri.it

**Keywords:** NSCLC, immunotherapy, prognostic, predictive, score

## Abstract

Background: Beyond programmed death ligand 1 (PD-L1), no other biomarkers for immunotherapy are used in daily practice. We previously created EPSILoN (Eastern Cooperative Oncology Group performance status (ECOG PS), smoking, liver metastases, lactate dehydrogenase (LDH), neutrophil-to-lymphocyte ratio (NLR)) score, a clinical/biochemical prognostic score, in 154 patients treated with second/further-line immunotherapy. This study’s aim was to validate EPSILoN score in a different population group. Methods: 193 patients were included at National Cancer Institute of Milan (second-line immunotherapy, 61%; further-line immunotherapy, 39%). Clinical/laboratory parameters such as neutrophil-to-lymphocyte ratio and lactate dehydrogenase levels were collected. Kaplan–Meier and Cox hazard methods were used for survival analysis. Results: Overall median progression-free survival and median overall survival were 2.3 and 7.6 months, respectively. Multivariate analyses for Progression-Free Survival (PFS) identified heavy smokers (hazard ratio (HR) 0.71, *p* = 0.036) and baseline LDH < 400 mg/dL (HR 0.66, *p* = 0.026) as independent positive factors and liver metastases (HR 1.48, *p* = 0.04) and NLR ≥ 4 (HR 1.49, *p* = 0.029) as negative prognostic factors. These five factors were included in the EPSILoN score which was able to stratify patients in three different prognostic groups, high, intermediate and low, with PFS of 6.0, 3.8 and 1.9 months, respectively (HR 1.94, *p* < 0.001); high, intermediate and low prognostic groups had overall survival (OS) of 24.5, 8.9 and 3.4 months, respectively (HR 2.40, *p* < 0.001). Conclusions: EPSILoN, combining five baseline clinical/blood parameters (ECOG PS, smoking, liver metastases, LDH, NLR), may help to identify advanced non-small-cell lung cancer (aNSCLC) patients who most likely benefit from immune checkpoint inhibitors (ICIs).

## 1. Introduction

Immune checkpoint inhibitors (ICIs), especially anti- programmed death receptor-1 (PD-1)/programmed death-ligand 1 (PD-L1) inhibitors, have significantly improved the therapeutic scenario of advanced non-small-cell lung cancer (aNSCLC) [1]. Nivolumab, pembrolizumab and atezolizumab have been approved in pretreated aNSCLC patients, in both squamous and nonsquamous histology, based on a significant improvement in overall survival (OS) versus docetaxel [2,3,4,5]. Pembrolizumab is currently the standard of care as first-line therapy in PD-L1 >50% aNSCLC [6]. Recently, combinations of ICIs with platinum-doublet chemotherapy (Keynote-189 and Keynote-407) and bevacizumab (IMpower150), as well as the association of nivolumab with Anti- Cytotoxic T-Lymphocyte Antigen 4 (anti-CTLA-4) agent ipilimumab (CheckMate 227), have shown survival benefit compared to standard chemotherapy, emerging as new treatment options for first-line setting in aNSCLC [7]. The introduction of these novel treatments in the clinical practice has generated several challenges, including the evidence of novel patterns of response and the management of new adverse events [8,9,10]. Despite the survival benefit obtained with ICIs, only a proportion of patients respond to immunotherapy and/or experience a durable clinical benefit [11]. The identification of predictive and/or prognostic biomarkers essential to identify patients most likely to respond to immunotherapy is a crucial point of ongoing clinical trials. Tumor PD-L1 expression is the only approved and most studied biomarker in aNSCLC, but it is limited by many biological and technical issues due to its intratumoral heterogeneity and temporal change expression [12]. Moreover, its predictive role remains unclear as low or negative PD-L1 are also shown to respond to immunotherapy [12]. Nowadays, clinical characteristics are the only selection parameters to determine candidate patients for immunotherapy [13]. Recently, other potential biomarkers have been investigated, such as tumor mutation burden (TMB), immune-score, cluster of differentiation 8 (CD8)-positive tumor-infiltrating lymphocytes and immune gene signature, but, to date, none have gained a definite role in clinical practice [12]. Peripheral blood inflammatory parameters have been investigated as potential cancer inflammation-associated markers and have shown to correlate with poor prognosis and lower response to standard treatments in various malignancies, including NSCLC [14]. They have been evaluated in advanced melanoma and aNSCLC patients receiving ICIs, especially for their prognostic role, while few data on their predictive role are reported [15]. 

Formerly, we investigated a prognostic score (EPSILoN score—ε score) based on five blood parameters and clinical characteristics (Eastern Cooperative Oncology Group performance status (ECOG PS), smoking status, presence of liver metastases, lactate dehydrogenase levels (LDH) and neutrophil-to-lymphocyte ratio (NLR). This score was developed by a retrospective monocentric analysis of 154 aNSCLC patients receiving single-agent anti-PD-1 inhibitors as ≥second line therapy [16]. The score was able to identify three different prognostic survival groups [16].

The aim of this study is to validate ε score in a different population of patients treated with immunotherapy in the same setting. 

## 2. Results

### 2.1. Patients’ Characteristics, Response and Survival Outcome

One hundred ninety-three aNSCLC patients treated with single-agent anti-PD 1 or anti PD-L1 in second- and further-line were included. Patients’ characteristics are summarized in Table 1. 

Most patients were male (62%) and smokers (80%); median age was 65 years (range 30–88 years) with 32% patients ≥70 years. Median Eastern Cooperative Oncology Group performance status (ECOG PS) was 1 (range 0–2) with an ECOG PS 2 in 12% of patients. All patients had histological diagnosis of NSCLC (23% squamous and 77% nonsquamous) and were Epidermal Growth Factor Receptor (EGFR) non-mutated and Anaplastic Lymphoma Kinase (ALK) non-translocated. 

At the time of ICIs start, bone metastases were present in 45% of patients, central nervous system (CNS) metastases in 23% of patients and bone liver metastases in 20% of patients. One hundred eighteen patients (61%) received immunotherapy in second line, while 75 patients (39%) received anti-PD-1 therapy in third- and further-line. Response and survival were evaluable for all 193 patients included in the study. At the time of data cut-off (April 2019), 179 patients (93%) had disease progression and 159 patients were dead (82%). After a median follow-up of 43.3 months (95% CI 40.3–46.5 months), median progression-free survival (mPFS) was 2.3 months (95% CI 1.9–2.6 months) and median OS (mOS) was 7.6 months (95% CI 5.4–9.9 months). Objective response rate (ORR) and disease control rate (DCR) were 18% (95% CI 12.6–23.9) and 44% (95% CI 36.4–50.8), respectively.

### 2.2. Survival Analysis According to EPSILoN Score

At univariate and multivariate analyses according to PFS adjusted for age, sex, smoking status, ECOG PS, histology and number of disease sites, heavy smoking status (≥40 pack/years) (HR 0.71, *p* = 0.036) and baseline LDH < 400 mg/dL (HR 0.66, *p* = 0.026) were confirmed as independent positive prognostic factors. On the other hand, baseline ECOG PS 2 (HR 1.79, *p* < 0.001), presence of liver metastases at baseline (HR 1.48, *p* = 0.04) and NLR ≥ 4 (HR 1.49, *p* = 0.029) were confirmed as independent negative prognostic factors (Table 2). The five variables were combined to define the three categories of the ε score and patients were stratified accordingly. Twenty-four patients (12%) were assigned to the favorable (group 1), 117 (61%) to the intermediate (group 2) and the remaining 54 patients (27%) to the poor category (group 3).

**Figure 1:**
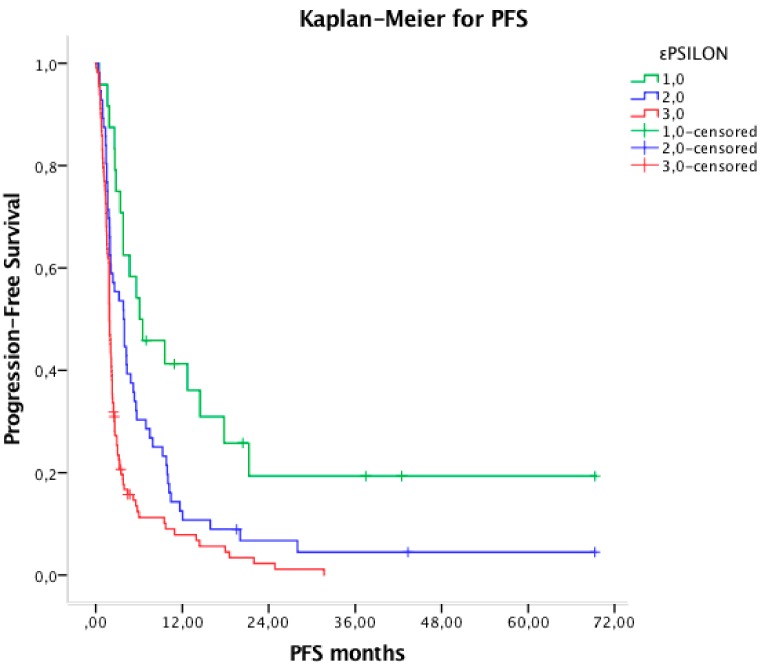
Kaplan–Meier curve for PFS dividing patients in three different prognostic groups.

**Figure 2:**
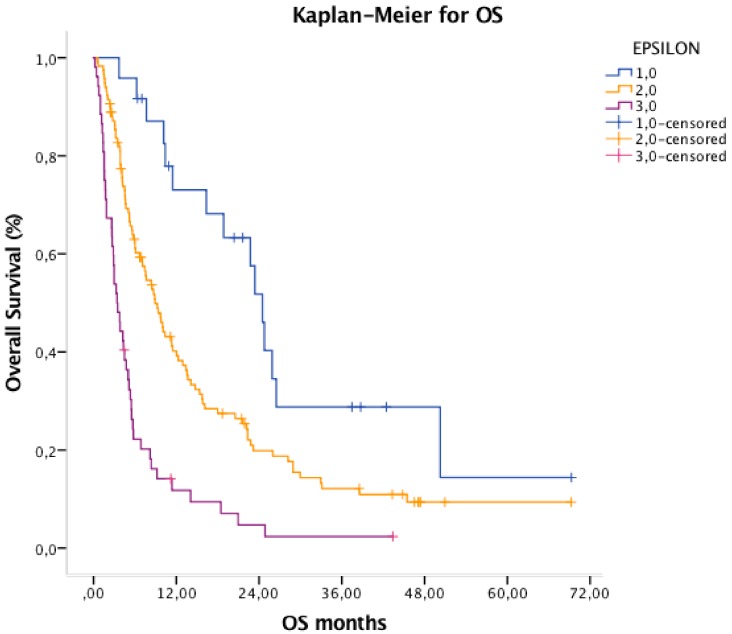
Kaplan–Meier curve for Overall Survival (OS) dividing patients in three different prognostic groups.

Median PFS were 6.0, 3.8 and 1.9 months for the favorable group 1, the intermediate group 2 and of the poor group 3, respectively (HR 1.94, 95% CI 1.51–2.48, *p* < 0.001) (Figure 1). Median OS of the three prognostic groups were 24.5, 8.9 and 3.4 months, respectively (HR 2.40, 95% CI 1.82–3.17, *p* < 0.001) (Figure 2).

## 3. Discussion

Immunotherapy has significantly improved the therapeutic landscape of aNSCLC, increasing long-term survival [1]. However, a small number of patients respond to ICIs both in section- and first-line monotherapy in daily practice (about 25–30%) [1,17]. Moreover, the association of chemotherapy plus immunotherapy improved response and survival outcomes in the first-line setting, but toxicity rates doubled due to the addition of chemotherapy [7].

The identification of prognostic and/or predictive biomarkers in order to recognize potential responders to anti-PD-1/PD-L1 inhibitors is deeply needed. The early identification of nonresponders could avoid inadequate treatments, unnecessary toxicity and high costs [18]. According to clinical factors, there is no agreement on the advantage of ICIs in a specific clinical subcategory of patients. Similar to other trials [19,20,21,22], our retrospective study has emphasized the negative prognostic role of ECOG PS 2, never-smoker status and presence of liver metastases in aNSCLC patients treated with ICIs. 

A poor ECOG PS leads to a reduced benefit from ICIs probably due to a frailer immune system with less functional lymphocytes and a short life expectancy. Hence, ECOG PS 2 patients have been usually excluded from ICIs trials and they are also underrepresented in studies specifically designed for special populations not generally included in clinical trials [23]. More data are awaited from ongoing prospective studies assessing the efficacy of immunotherapy (NCT02733159, NCT02879617) in ECOG PS 2 NSCLC patients [24,25]. Whether ECOG PS is a prognostic and/or predictive biomarker in patients treated with ICIs remains an open question so far. 

Numerous trials showed that patients who were former/current smokers benefited more from ICIs compared to nonsmokers [1,26,27,28]. Smoking-related NSCLC was generally associated with high PD-L1 expression and high TMB levels, resulting in a greater expression of neoantigens able to foster anticancer immune response upon ICI treatment. 

Immunotherapy-related survival outcomes correlated with type of metastases at baseline ICIs are unknown. However, some studies revealed that ICI efficacy varies based on different metastatic sites [18,29]. This organ-specific response may be the result of the different PD-L1 expression, microenvironment and genetic heterogeneity profiles between primary and metastatic sites. Many retrospective analyses on NSCLC and melanoma patients with liver metastases, treated with ICIs, experienced notably poorer response rates and survival outcomes [30,31]. The liver is characterized by an immune-suppressive microenvironment where IL-10-secreting dendritic cells, Kupffer macrophages and sinusoidal endothelial cells may induce T-cell anergy and decreased likelihood of response to immunotherapy alone [32,33]. However, these patients have shown an improvement in survival with the ICIs plus chemotherapy combination as compared to chemotherapy alone [34]. Recently, to boost the ICIs’ role, the addition of antiangiogenetic drugs to the ICIs plus chemotherapy combination have proven to result in a better outcome; probably, for these patients, this would the best treatment choice [32,33,35]. Our analysis showed the negative prognostic role of liver metastases, while the survival impact of bone and brain metastases remains still uncertain. Brain metastases are not confirmed as a negative prognostic factor because ICIs may cross the blood–brain barrier and induce disease response in selected patients [31]. Data from both retrospective series [36] and clinical trials showed good activity of ICIs as a single agent [37] or in combination with chemotherapy [38] in patients with brain metastases. 

In addition to clinical features, peripheral immune cells and inflammatory factors have also been recently explored as possible biomarkers of response to ICIs in melanoma, lung cancer and other types of tumors treated with ICIs [12,39,40,41,42,43,44,45].

Many studies investigated the prognostic value of NLR since it better reflects the equilibrium between protumor and antitumor activity of the host immune system [46]. NLR has been studied in patients treated with ICIs in different types of cancers and several thresholds have been proposed [47,48,49]. Higher derived NLR was associated with poor survival outcome [50] and hyperprogressive disease [51] in NSCLC patients treated with ICIs. A meta-analysis including 14 retrospective analyses and 1225 patients suggested that NLR may have a prognostic role in NSCLC patients receiving nivolumab [52]. However, derived NLR can be also prognostic for cytotoxic chemotherapy [53] or EGFR-Tyrosine Kinase Inhibitor (TKI) treatment [54]. Interestingly, neutrophils dominate NSCLC tumor microenvironment [55], and circulating cluster of differentiation 16 (CD16) low (i.e., immature) neutrophils correlated with fast progression upon ICI treatment in NSCLC [56] suggest that the characterization of phenotype and functional properties of circulating and intratumoral neutrophils are the next challenges in identifying specific neutrophil subpopulations as predictive factors for ICI therapies. Regarding LDH, it has been integrated in various scores [50] and its prognostic role may be due to the correlation with high tumor burden and increased hypoxia [57]. High LDH seems to negatively correlate with cytotoxic T lymphocyte activation, probably due to the inability of CD8 T cells to export lactate in the presence of a high extracellular concentration of tumor-derived lactic acid which impairs aerobic glycolysis [58]. 

The evaluation of different biomarkers in a single prognostic score rather than focusing on a single biomarker permits to better identify patients who most likely will (or will not) benefit from ICIs [13]. Many immune-based prognostic scores were studied using clinical features and blood biomarkers such as lung immune prognostic index (LIPI) [50], advanced lung cancer inflammation index (ALI) [59], immunotherapy sex-ECOG-NLR-delta NLR (iSEND) [60], systemic inflammation index (SII) [18,61] and aggregate index of systemic inflammation (AISI) [61]. All these scores incorporated NLR and most of them included ECOG PS and LDH. In addition, similar to ours, these were generated from retrospective analyses with a median number of 159 patients (range 54–466 patients), which is close to the number of patients included in our training cohort (*n* = 154) and in the current validation cohort (*n* = 193).

Another weakness of our study (as in other similar studies) is the absence of a control arm leading to the conclusion that all these scores are prognostic rather than predictive for immunotherapy. The LIPI score from Mezquita et al. was the only one with a validation set of patients treated with ICIs and a control cohort of patients treated with chemotherapy alone in the same setting [50]. Moreover, some of the reported scores were not associated with survival at baseline [50,59,61] but only at time-series analysis (i.e., at 6 weeks of treatment) [18]. 

Another potential weakness regards the applicability of ε score after the introduction in the clinical practice of first-line ICI-based combinations [1,62]. However, the variables of ε score are well-known prognostic markers also in aNSCLC patients treated with chemotherapy with or without bevacizumab in the first line [63,64,65,66]. Hence, in addition to the results obtained with immunotherapy alone, the ε score has the rationale to be assessed in first-line setting combinations in the near future. Furthermore, in some countries the access to first line ICI-based combinatorial treatment strategies in NSCLC will not be easily and shortly incorporated in clinical practice, especially for patients with high PD-L1 expression. Moreover, investigation of novel biomarkers is crucial to finding new target therapies that are able to overcome potential unknown mechanisms of resistance to current treatment approaches. For instance, some recent studies have investigated the CCDC6 protein which is downregulated in 30% NSCLCs, resulting in a defect of homologous recombination repair. In the contest of BReast CAncer gene (BRCA)-mutated cancers with compromised HR repair, low CCDC6 protein levels can increase lung cancers cells’ sensitivity to olaparib alone or synergize with chemotherapy. On the other hand, these DNA damaging agents can also increase the immunogenicity of the tumor and possibly improve ICI efficacy [67,68]. 

Despite all the limits mentioned above, the major strengths of our ε score are the inclusion of both clinical and blood markers and the relatively high number of patients included in the analysis. Moreover, in consideration of the routine assessment of these peripheral blood biomarkers, ε score could be easily and rapidly integrated into clinical practice, helping clinicians in the decision-making process. 

## 4. Materials and Methods

This study was conducted at one single institution in Italy (Fondazione IRCCS Istituto Nazionale Tumori of Milan) and was accomplished in agreement with Good Clinical Practice, Declaration of Helsinki, and local ethical guidelines. The trial was approved by the local ethical committee of Fondazione IRCCS Istituto Nazionale dei Tumori of Milan (Trial No. INT 22-15). All living patients enrolled in the study signed the informed consent.

### 4.1. Study Population, Treatment and Response Evaluation

We retrospectively collected clinical data and laboratory parameters of 193 consecutive aNSCLC (stages IIIb–c and IV) patients treated with single-agent anti-PD-1/PD-L1 inhibitors in second- or further-line therapy from August 2013 to April 2019. Treatment was continued until progressive disease (PD), intolerable toxicity, patient’s withdrawal of consent or death. Treatment beyond PD was allowed according to physicians’ decision and in presence of clinical benefit. Patients treated with first-line ICIs single-agent or in combination with chemotherapy or other systemic drugs were excluded from the analysis.

Response was assessed by computed tomography scan performed baseline and every 3–4 cycles (~12 weeks), or whenever PD was clinically suspected. Response was evaluated by Response Evaluation Criteria in Solid Tumors (RECIST) v.1.1 [69]. ORR was defined as the sum of complete response (CR) and partial response (PR). DCR results as the sum of CR, PR and stable disease (SD).

### 4.2. Statistical Analysis

The primary objective was to determine whether ε score was able to categorize patients into three different survival groups. PFS was defined as the time from ICIs start date to PD or death from any cause, while OS was defined as the time from ICIs start date to death from any cause or last follow-up. Survival curves were estimated by Kaplan–Meier method and compared by log-rank Mantel test. All *p*-values were two sided, and values less than 0.05 were considered statistically significant. Patients were categorized in three scoring groups (favorable, 0; intermediate, 1–2; poor, 3–5) according to the scoring system previously generated (Table 3) [16]. Optimal cut-offs for LDH and NLR values were determined using a statistic method which enables calculation of both the cut-off value and its significance [70]. We applied the method used by Newcombe et al. [71] to calculate two-sided confidence intervals for the single proportion. Cox progression hazard model was used for multivariate analyses and to compare survival outcomes according to the three ε categories.

Statistical analyses were implemented using the Statistical Package for the Social Sciences (SPSS) program version 25.0 (IBM, Armonk, NY, USA) [72].

## 5. Conclusions

In conclusion, we have previously generated and here validated a baseline prognostic score (EPSILoN, ε score—ECOG PS, smoking, liver metastases, LDH and NLR) for aNSCLC patients treated with second- or further-line single-agent ICIs. This score statistically significantly identifies three different prognostic groups of patients and could be a useful tool to guide treatment decisions. 

## Figures and Tables

**Table 1:** Patients’ characteristics at baseline immunotherapy.

Validation Cohort	Characteristics *n* (%) *n* = 193
Gender Male Female	120 (62) 73 (38)
Median age, years (range) <70 ≥70	65 (30–88) 131 (68) 62 (32)
ECOG PS Median (range) 0 1 2	1 (0–2) 68 (36) 99 (52) 23 (12)
Smoking status: Former/current smoker Never smoker	150 (78) 43 (22)
Median pack years, (range)	30 (0–177)
Histologic subtype Adenocarcinoma Squamous Other histologies	144 (74) 44 (23) 5 (3)
Stage IIIb–c IV	5 (3) 188 (97)
Liver metastases Yes No	38 (20) 155 (80)
Bone metastases Yes No	87 (45) 106 (55)
Brain metastases Yes No	44 (23) 149 (77)
Treatment line Second line ≥Third line	118 (61) 75 (39)

ECOG PS, Eastern Cooperative Oncology Group Performance Status.

**Table 2:** Multivariate analyses for progression-free survival (PFS) using Cox progression hazard model.

Variables	HR	95% CI for HR (Range)		*p*-Value
Age <70 y ≥70 y	0.83	0.59	1.18	0.299
Sex Male Female	1.06	0.76	1.47	0.736
Smoke ≥40 p y <40 p y	0.72	0.52	0.98	0.036
Histology non-Sq Sq	0.99	0.66	1.48	0.962
ECOG PS 0/1 2	1.78	1.38	2.29	<0.001
No. Disease Site ≥3 <3	1.12	0.94	1.33	0.200
Liver mets	1.48	1.12	2.09	0.044
CNS mets	0.87	0.57	1.32	0.507
Bone mets	0.97	0.67	1.41	0.869
NLR ≥4 <4	1.49	1.04	2.14	0.029
LDH <400 mg/dL ≥400 mg/dL	0.66	0.46	0.95	0.026

p y, pack years; y, years; sq, squamous; non-sq, nonsquamous; ECOG PS, Eastern Cooperative Oncology Group Performance Status; mets, metastases; CNS, Central Nervous System; NLR, Neutrophil-to-Lymphocyte Ratio; LDH, Lactate Dehydrogenase.

**Table 3:** Baseline predictive score: EPSILoN (ECOG PS, smoking, liver metastases, LDH, NLR; ε score).

Prognostic Factor	Assessment	Point
ECOG PS	1 2	0 1
Smoking (pack years)	≥40 <40	0 1
Liver metastases	No Yes	0 1
LDH (mg/dL)	<400 ≥400	0 1
NLR	<4 ≥4	0 1
**Prognostic groups** (points): best = 0 intermediate = 1–2 poor = 3–5		

ECOG, Eastern Cooperative Oncology Group; PS, performance status; LDH, lactate dehydrogenase; NLR, neutrophil-to-lymphocyte ratio.
